# WhatsApp-Based Coaching Program to Support Smoking and Vaping Cessation Among Young People: Pre-Post Study on Acceptance and Preliminary Efficacy

**DOI:** 10.2196/65301

**Published:** 2025-10-02

**Authors:** Severin Haug, Lisa Caputo, Andreas Wenger, Nikolai Kiselev, Olivia Studhalter, Michael P Schaub

**Affiliations:** 1 Swiss Research Institute for Public Health and Addiction, University of Zurich, Konradstrasse 32, Zurich, 8005, Switzerland, 41 0444481174

**Keywords:** smoking cessation, vaping, nicotine, mobile phone, WhatsApp, young people, adolescents

## Abstract

**Background:**

The use of tobacco cigarettes and electronic nicotine products is widespread among young people in Switzerland. At the same time, the instant messaging platform WhatsApp (Meta Platforms, Inc) is the most frequently used smartphone app in this population group. The provision of individually tailored, evidence-based coaching messages via WhatsApp seems promising to support smoking cessation in adolescents and young adults.

**Objective:**

This study aims to test the feasibility, acceptance, and preliminary efficacy of a newly developed, semiautomated WhatsApp-based intervention program to support smoking and vaping cessation and reduction in adolescents and young adults.

**Methods:**

Recruitment took place in Switzerland in 2023 and 2024 via various channels, both online and offline. For a period of 11 weeks, regular users of cigarettes or electronic cigarettes, aged between 16 and 30 years, received individually tailored messages on how to deal with cravings or stressful situations and how to stop or reduce smoking. A separate WhatsApp channel provided the opportunity to ask individual questions to a counselor. A one-group pre-post design was used to obtain preliminary information on the acceptability and potential efficacy of the program.

**Results:**

A total of 167 young people (mean age 23.2, SD 4.0 years; n=95, 56.9% women and n=72, 43.1% men) who regularly smoked tobacco cigarettes (n=81, 48.5%), vaped electronic nicotine products (n=17, 10.2%), or used both (n=69, 41.3%) were recruited for participation in the program. Of these, 100 (59.9%) intended to stop smoking or vaping while 67 (40.1%) aimed at reducing their use. The participants actively engaged in an average of 5.5 (SD 3.5) of the 11 program weeks, the average number of interactions with the program was 26.8 (SD 26.1), and the average duration from the start of the program to the last interaction was 45.0 (SD 31.1) days. The follow-up survey at the end of the 11-week coaching program was completed by 108 (64.7%) participants. The generalized estimating equation (GEE) analyses revealed significant reductions in the mean number of days in the last 30 days on which tobacco cigarettes were used from 20.6 (SD 11.8) at baseline to 14.0 (SD 12.0) at post assessment (incidence rate ratio [IRR] 0.68, *P*<.001) and for electronic nicotine products from 11.1 1 (SD 13.1) days at baseline to 7.7 (SD 11.3) days at follow-up (IRR 0.71, *P*=.005). Overall, 6/108 (5.6%) participants in the follow-up survey stated that they neither consumed tobacco cigarettes nor electronic nicotine products in the last 30 days.

**Conclusions:**

The WhatsApp-based program appears to be a feasible, moderately accepted, and promising intervention for reducing the consumption of tobacco cigarettes and electronic nicotine products among young people. A larger-scale randomized controlled trial would be reasonable in order to make more substantiated statements about the program’s efficacy.

## Introduction

Nicotine and tobacco use is a major cause of the disease burden in most countries of the world [1]. Every year, it causes 7.7 million deaths worldwide and more than 9000 deaths in Switzerland, with the majority of tobacco-related deaths due to noncommunicable chronic diseases, such as cardiovascular diseases, respiratory diseases, and tumors [2,3]. Despite the well-documented health risks, many young people, that is, adolescents and young adults, start smoking or experiment with smoking. In Switzerland, 27% of men and 21% of women are regularly smoking tobacco, with the highest prevalence rate of 30% among young adults between the ages of 20 and 24 years [4]. Young people should therefore be at the center of preventive and early interventions, particularly because quitting smoking before the age of 30 years can prevent more than 97% of the excess mortality caused by continued smoking [5].

Although the consumption of tobacco cigarettes has declined in many European countries in recent years, there has been an increase in the consumption of electronic nicotine products, such as electronic cigarettes (e-cigarettes), also known as vapes, particularly among adolescents and young adults between the ages of 15 and 24 years [6]. There is substantial evidence that e-cigarettes can aid in smoking cessation for adults [7] and that e-cigarettes may be less harmful than cigarettes [8]; however, there is currently insufficient scientific evidence to fully understand the short- and long-term health effects of these electronic nicotine products [7]. They are not harm-free, particularly for adolescents, as vaping is associated with similar nicotine metabolite levels compared with smoking tobacco [9]. Furthermore, there is substantial evidence that electronic nicotine products can cause addiction and that young nonsmokers who use these products are more likely than nonusers to initiate tobacco smoking [10,11].

There is extensive literature demonstrating the effectiveness of traditional behavioral counseling and pharmacological interventions for smoking cessation in adults [12-14]; however, there is only little evidence regarding their effectiveness in adolescents and young adults [15]. Furthermore, their widespread implementation faces barriers, and they are only used by a minority of young people with a high motivation to quit [16,17].

Mobile phone–based interventions have the potential to reach young smokers and vapers who are not or only partially reached by traditional interventions, owing to their 24/7 availability and anonymity [18]. A review on mobile phone–based smoking cessation interventions among young people demonstrated the efficacy of text messaging–based interventions [17], while the evidence regarding the effectiveness of smartphone app–based interventions is currently inconclusive [17,19]. In recent years, social media–based interventions have been increasingly developed and shown to be a promising way of delivering smoking cessation content to young people [20,21]. Social media–based smoking cessation interventions that use platforms like Facebook (Meta Platforms, Inc), TikTok (ByteDance), or WeChat (Tencent) typically provide peer support, individually tailored content, and community engagement, while traditional text message–based interventions deliver more structured, automated, and individually tailored messages. A randomized controlled trial with young adults from the United States investigated the effectiveness of a Facebook-based smoking cessation program and revealed significant short-term effects on abstinence [22]. In a controlled trial with adult Chinese smokers, the effectiveness of a WeChat-based smoking cessation intervention was demonstrated [23].

To date, no studies have been conducted on the acceptance or effectiveness of automated WhatsApp-based smoking cessation programs. Nevertheless, WhatsApp has characteristics that make it a promising platform for delivering smoking and vaping cessation interventions. In Switzerland, as in many other European countries, WhatsApp is the most widely used instant messenger and the most used smartphone app among adolescents and young adults [24]. Similar to SMS text messaging, WhatsApp is a regularly used communication channel that requires neither familiarization with the functionality of a new platform nor the opening of a standalone app. In addition, WhatsApp offers significant advantages over text messaging, including the ability to directly incorporate media objects and the option to send messages free of charge with an existing internet connection.

The objectives of this study were (1) to test the feasibility of a WhatsApp-based smoking and vaping cessation intervention, (2) to investigate its acceptance among adolescent and young adult nicotine users, and (3) to provide a test of its preliminary efficacy.

## Methods

### Intervention Program

#### Technological Background

The rauchfreiCoach (English translation: SmokefreeCoach) intervention program was developed in 2022 and 2023 by the Swiss Research Institute for Public Health and Addiction (Institut für Sucht- und Gesundheitsforschung; ISGF) and PathMate Technologies as a German-language program for use in the German-speaking part of Switzerland. SmokefreeCoach is the world’s first automated WhatsApp-based program to promote smoking and vaping cessation among adolescents and young adults.

**Figure 1.**
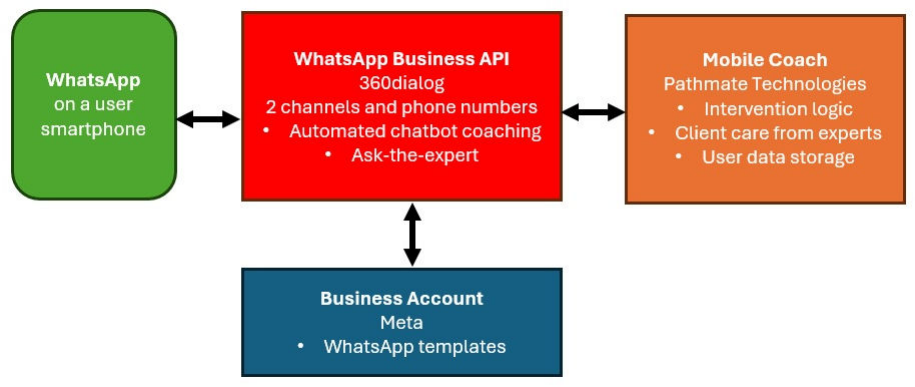
Software architecture of SmokefreeCoach. API: application programming interface.

The SmokeFreeCoach consists of 4 essential components; refer to Figure 1. The first component is a participant using a smartphone with WhatsApp installed. The second component is a business solution provider who provides a WhatsApp Business API (application programming interface). Facebook launched this API in 2018 but only for certified partners, which was 360dialog in our case. The third component is a business account with Meta by which one can create message templates. Those templates are needed to initiate a dialog with a user. The fourth component is a technological partner who realizes the actual intervention. In our case, this was PathMate Technologies, which ran the intervention logic, provided a web interface to chat with the participants, and stored the scientific data.

#### Intervention Content

The program’s target group was young people between the ages of 16 and 30 years who consumed tobacco cigarettes or electronic nicotine products at least once a week and used WhatsApp on their smartphones. The program was based on principles from Prochaska and DiClemente’s transtheoretical model [25] and Bandura’s social cognitive theory [26]. In addition, SmokefreeCoach applied various techniques that, according to recent literature reviews [18,27], were promising for increasing the use of digital interventions and promoting the desired behavioral changes. These include the individualization of content, individual support from a professional, regular coaching messages, ease of use and technical stability, consideration of personally relevant goals, regular monitoring of goal achievement, providing information on the health risks associated with smoking, weighing up the pros and cons of quitting smoking, and highlighting alternative behaviors. The program was available in 3 versions, whose contents differed depending on the individual’s specified behavioral goal. Version 1 was designed for participants who neither wanted to quit nor wanted to smoke or vape less, version 2 for participants who wanted to reduce their consumption, and version 3 for participants who wanted to stop smoking or vaping. Participants had the possibility to modify their behavioral goal at any time during program runtime. For participants who regularly smoked tobacco cigarettes and vaped electronic nicotine products, the specified goal applied to both products.

Table 1 displays the intervention program elements according to the individual consumption goal. Due to the optional on-demand tips and the higher-intensity quit program, the intervention dose was very heterogeneous, ranging from 1 message per week for participants without a reduction or cessation goal and no use of on-demand tips to 3 messages per day for participants with a cessation goal and an activated higher-intensity quit program.

**Table 1.** Intervention program elements according to the selected consumption goal.

Intervention program element	Neither reduction nor cessation goal	Reduction goal	Cessation goal
Stress management tips tailored to individually indicated stressors	✓	✓	✓
Motivational messages tailored to individually indicated benefits of smoking or vaping cessation or reduction	✓	✓	✓
On-demand tips on motivation, stress, and craving	✓	✓	✓
Ask-the-Expert	✓	✓	✓
Strategies to deal with individually indicated barriers and obstacles during smoking or vaping reduction		✓	
Monitoring of goal achievement, that is, smoking or vaping cessation in self-selected trigger situations		✓	
If-then behavior plans and strategies to refrain from smoking or vaping in self-selected trigger situations		✓	
Strategies to deal with individually indicated barriers and obstacles during smoking or vaping cessation			✓
Monitoring of goal achievement, that is, the number of consumption days in the previous week			✓
If-then behavior plans and strategies to refrain from smoking or vaping in self-selected trigger situations			✓
Higher-intensity quit the program around the self-selected quit date			✓

#### Program Flow

**Figure 2.**
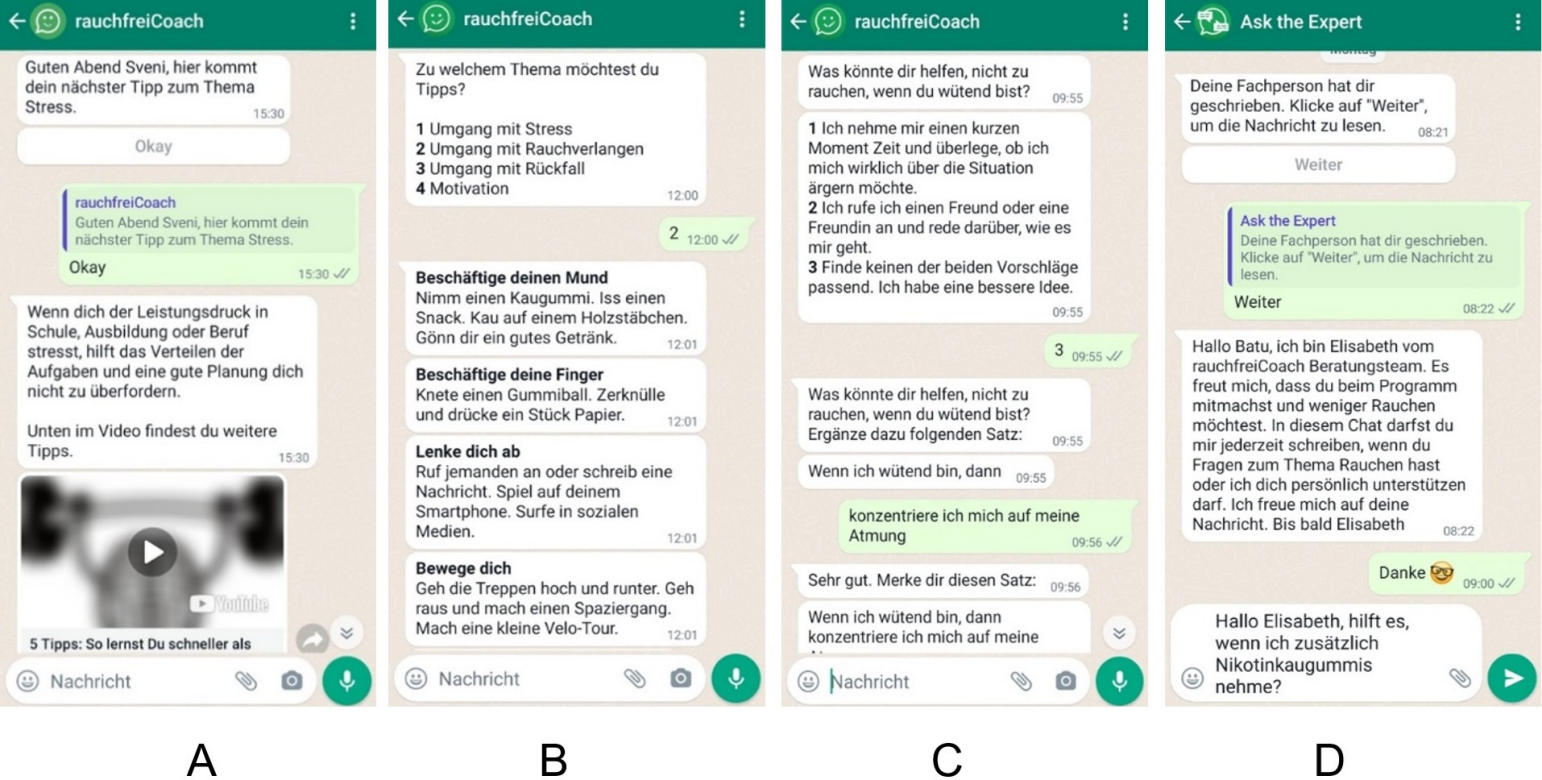
Sample screenshots of the program (from left to right): (A) individual tip to cope with performance pressure, (B) on-demand tips to cope with craving for cigarettes, (C) if-then behavior plan on how not to smoke when feeling angry, and (D) Ask-the-Expert channel to pose individual questions to a smoking cessation expert.

All participants, regardless of their consumption goal, received 2‐4 weekly WhatsApp messages that provided tips on how to deal with stress as well as motivational tips for a period of 11 weeks. Furthermore, all participants could access motivational tips and tips on dealing with stress, cravings, and relapse at any time. Through a separate WhatsApp channel, all participants also had the opportunity to ask individual questions to a smoking cessation expert anonymously and free of charge (Ask-the-Expert). This expert was a trained smoking cessation counselor from the Zurich Lung Association, that is, an external counselor who was not part of the research team. These individual questions were answered during working hours within a maximum of 2 working days. Figure 2 shows a selection of screenshots of the program (in German), which illustrate different types of conversation within the program.

In addition to the program elements already mentioned, participants with a reduction goal received weekly monitoring of goal achievement, that is, smoking or vaping cessation in certain situations, as well as WhatsApp messages that helped them with strategies to stop smoking or vaping in these selected situations. Participants who intended to quit received weekly monitoring of their goal achievement by assessing the consumption days in the previous week as well as messages helping them with strategies to stop smoking or vaping. Participants who were determined to give up smoking or vaping could also sign in to a higher intensity quit program. This optional component of the program started 7 days before an individually selected quit date and accompanied the participants with an additional 2 messages per day for cessation preparation and relapse prevention until 14 days after the self-selected quit date.

### Study Design and Procedure

A longitudinal pre-post study design was used to test the feasibility, acceptance, and preliminary efficacy of the program. In order to identify small to medium effect sizes, we planned to recruit at least 100 people for this pre-post pilot study. Study participants were recruited between May 26, 2023, and March 12, 2024. The program and the associated website were advertised via various channels in the German-speaking part of Switzerland. These included unpaid posts on an Instagram (Meta Platforms, Inc) channel, an announcement in the Swiss free newspaper 20 Minuten*,* announcements in the mailing list of the University of Zurich, information for vocational school teachers and vocational trainers about the program, and a market stall at the Zurich University of Applied Sciences. In addition, the program was promoted through personal contacts with colleagues, family members, friends, and acquaintances of the Zurich Center for the Prevention of Substance Abuse (Zürcher Fachstelle zur Prävention des Suchtmittelmissbrauchs; ZFPS) and the ISGF.

People interested in the program were asked to register using the sign-in button or QR code. Subsequently, the inclusion criteria for study participation (ages between 16 and 30 years and at least weekly consumption of tobacco or electronic nicotine products in the previous 4 weeks) were assessed and checked within a brief WhatsApp dialog. If the inclusion criteria were met, detailed information on the program, its aims, and the associated study was provided. After agreeing to participate in the program and the related study within the WhatsApp dialog via tickbox, participants were asked to select a username and to complete the baseline survey (refer to “Measures and Outcome Criteria” below).

The 10-week coaching always began on the first Monday after registration. During the period between registration and the actual start of coaching, which could last from 1 to 7 days, participants already had the opportunity to interact with the program. Therefore, the total duration of the program was 10 weeks plus 1‐7 days.

**Figure 3.**
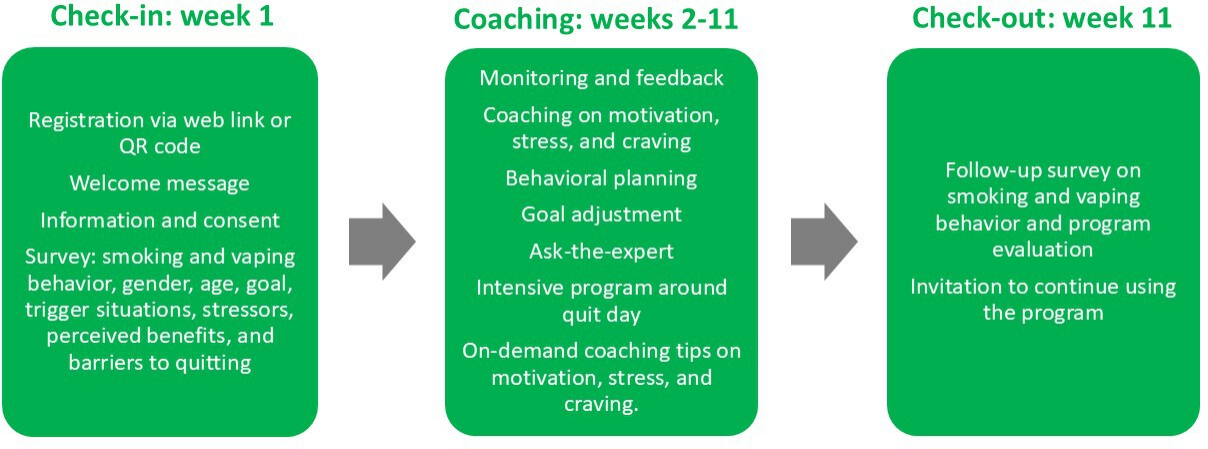
Procedure of the program and the study.

At the end of week 11, participants were invited via the WhatsApp channel to complete an online follow-up survey (refer to “Measures and Outcome Criteria”). If they did not do so, they were contacted again via an SMS text message and finally by phone call. Following the follow-up survey, participants had the opportunity to continue using the program. Figure 3 illustrates and summarizes the course of the program and the study.

### Measures and Outcome Criteria

The baseline survey included the following variables: (1) gender, (2) canton of residence, (3) recruitment channel, (4) the consumption goal (quitting, reducing, or neither quitting nor reducing), (5) emotional states that often tempt participants to smoke or vape, (6) situations in which participants smoke or vape frequently, (7) situations in which participants would like to stop smoking or vaping in the future, (8) current sources of stress and strain, (9) potential benefits of quitting or reducing nicotine use, and (10) participants’ concerns about quitting or reducing. Items (5) to (10) were multiple-response items, derived from the current literature on nicotine and tobacco use in young people and consolidated within 2 focus groups with 8 and 7 adolescents and young adults, who regularly smoked or vaped nicotine products. Beyond consolidating the response items, the focus groups aimed at optimizing the program flow, the logo, and the integrated images and illustrations.

Variables that were assessed in both the baseline and follow-up survey and used as outcome criteria included (1) the number of days in the past 30 days smoking tobacco cigarettes, (2) the number of tobacco cigarettes smoked per smoking day, and (3) the number of days in the past 30 days using electronic nicotine products (eg, e-cigarettes, electronic shisha). By using (1) and (2), we calculated the average number of tobacco cigarettes consumed per day in the last 30 days. Participants who indicated that they did not smoke tobacco or electronic nicotine products in the past 30 days in the follow-up survey were considered as nonsmokers.

To assess program use and acceptance, we also assessed in the follow-up survey whether participants (1) had usually read the program messages thoroughly, (2) had usually only looked at the program messages briefly, or (3) had not read the program messages. Participants also had to indicate whether the content and tips of the program were (1) understandable, (2) helpful, (3) adapted to their personal situation, (4) motivating to smoke less or quit smoking, and (5) helpful to smoke less or quit smoking using the response options 1=thumbs up, 2=thumbs up and down, and 3=thumbs down. In addition, participants were asked to rate specific program elements (the form of coaching via WhatsApp, the tips received, the opportunity to ask questions to an expert, and the intensive quit program) using the response options, 1=thumbs up, 2=thumbs up and down, 3=thumbs down, and 4=can’t judge, as well as to give the program an overall rating on a scale of 1-5 stars. Finally, participants were asked to indicate which functions or content of the program they found particularly interesting or helpful and to make suggestions for improving the program. The detailed baseline and follow-up survey questions are available in Multimedia Appendix 1.

In addition to the variables recorded in the baseline and follow-up surveys, the following indicators of program engagement were also extracted from the program log files for each participant: (1) whether or not the participant posed individual questions in the Ask-the-Expert channel, (2) the number of active weeks, that is, weeks during which at least 1 coaching message was retrieved or requested by a participant, (3) the total number of retrieved and requested coaching messages of a participant, and (4) the number of days from the start of the program to the last interaction with the program. Interaction with the program was defined as any response by the participant to the messages automatically sent by the program or any spontaneous request by the participant to the program.

### Data Analyses

An attrition analysis was conducted to test for significant differences between participants who completed the follow-up survey and those who did not. Mann-Whitney *U* tests were used for non-normally distributed metric variables and chi-square tests for categorical variables.

We used generalized estimating equation (GEE) analyses to investigate the longitudinal course of the outcome criteria over the study period of 10 weeks. GEE is a repeated-measures regression model that takes into account the correlation of the repeated measures within a person [28]. It is a powerful and versatile procedure for analyzing longitudinal data under minimal assumptions about time dependence and allowed us to use all available longitudinal data, irrespective of single missing values at follow-up. We created separate GEE models for our outcomes: (1) number of days in the past 30 days smoking tobacco cigarettes, (2) number of days in the past 30 days using electronic nicotine products, and (3) average number of tobacco cigarettes consumed per day in the last 30 days. Each model contained the time variable as an independent variable (baseline vs follow-up survey), one of the three variables associated with the consumption of nicotine-containing products as a dependent variable, and all variables that proved to be significant in the attrition analysis as covariates. In this way, it was possible to control for attrition bias. As all participants were smokers at baseline assessment, it was not possible to develop a converging GEE model for the 30-day point prevalence abstinence rate, and we only provided descriptive statistics for this outcome.

The open-text data on helpful program elements and suggestions for improving the program were analyzed and presented in a quantitative-descriptive manner. Based on qualitative content analysis [29], a PhD-level scientist determined the level of abstraction of the categories in the first step. In a second step, the responses were assigned to one or more of the available categories. An alpha level of .05 (2-tailed) was chosen for all statistical tests in this study. The analyses were performed using the Stata version 12 (StataCorp LLC) software package, or SPSS version 29 (IBM Corp).

### Ethical Considerations

The study was approved by the Ethics Committee of the Faculty of Philosophy of the University of Zurich (approval date: December 20, 2021; approval number 21.12.15). Digital informed consent was obtained from the participants within the onboarding WhatsApp dialog. No additional parental consent was necessary for 16- and 17-year-olds. Study participants completed the screening procedure and baseline assessment anonymously. No names were collected, and participants instead chose nicknames. The business solution provider, which provided the API for WhatsApp communication to end users, ensured General Data Protection Regulation (GDPR) compliance. Data were transmitted with end-to-end encryption and were stored on a server in Switzerland. Participants received an incentive of 10 Swiss Francs (around US $12) for taking part in the follow-up survey.

## Results

### Study Participants

A total of 167 young people (mean age 23.2, SD 4.0 years; n=95; 56.9% women, n=72, 43.1% men) who regularly smoked tobacco cigarettes (n=81, 48.5%), vaped electronic nicotine products (n=17, 10.2%), or both (n=69, 41.3%) could be recruited for participation in the program and the associated study. Of these, 59.9% (n=100) intended to quit, while 40.1% (n=67) aimed at reducing their use of cigarettes or electronic nicotine products. None of the participants stated that they wanted neither to stop nor to reduce. Table 2 presents the baseline characteristics of the program participants.

**Table 2.** : Baseline characteristics of program participants (N=167).

Characteristic		Value
Recruitment channel (multiple answers), n (%)		
Social media		10 (6)
Advertising in a commuter newspaper		12 (7.2)
Friends or acquaintances		35 (21)
School or University		93 (55.7)
Work or vocational trainer		20 (12)
Gender, n (%)		
Men		72 (43.1)
Women		95 (56.9)
Diverse		0 (0)
Age (years), mean (SD)		23.2 (4.0)
Canton of residence, n (%)		
Zurich		99 (59.3)
Aargau		17 (10.2)
St. Gallen		11 (6.6)
Grisons		7 (4.2)
Other canton		26 (15.6)
Other country		7 (4.2)
Products consumed, n (%)		
Solely tobacco cigarettes		81 (48.5)
Solely electronic nicotine products		17 (10.2)
Both tobacco cigarettes and electronic nicotine products		69 (41.3)
Behavioral goal, n (%)		
Stop smoking or vaping		100 (59.9)
Reduce smoking or vaping		67 (40.1)
Neither stop nor reduce		0 (0)
Number of days in the last 30 days on which tobacco cigarettes were smoked, mean (SD)		20.8 (11.6)
Average number of tobacco cigarettes smoked per day, mean (SD)		7.4 (7.0)
Number of days in the last 30 days on which electronic nicotine products were consumed, mean (SD)		10.0 (12.7)

After week 11, 108/167 (64.7%) program participants completed the follow-up survey. The attrition analyses revealed that follow-up responders did not differ from nonresponders with respect to any of the baseline characteristics. However, nonresponders were active during fewer program weeks (mean 3.6, SD 2.6 vs 6.5, SD 3.6; *z*=–5.3; *P*<.001), had fewer interactions with the program (mean 15.7, SD 20.1 vs 32.9, SD 27.0; *z*=–4.8; *P*<.001) and showed a lower number of days from program start to last interaction with the program (mean 27.6, SD 27.7 vs 54.5, SD 28.8; *z*=4.9; *P*<.001). These engagement variables were included as covariates in the GEE models investigating the longitudinal course of the outcome criteria.

### Acceptance of the Intervention Program

#### Program Attrition

During the program, which lasted for 11 weeks, none of the 167 participants actively unsubscribed from the program or withdrew from study participation.

#### Program Use

Among the 167 program participants, the average number of active weeks was 5.5 (SD 3.5). A total of 16 (9.6%) participants were inactive in any program weeks, while 26 (15.6%) participants were active in all program weeks. In addition, 80 (47.9%) participants were active in more than half, that is, at least 6, of the 11 program weeks.

The average number of interactions with the program was 26.8 (SD 26.1), and the mean duration from the start of the program to the last interaction was 45.0 (SD 31.1) days.

In the follow-up survey, 38/108 (35.2%) participants stated that they had usually read the program messages thoroughly, 58 (53.7%) participants stated that they usually looked at them briefly, and 12 (11.1%) participants stated that they usually had not read the program messages.

In total, 8/167 (4.8%) program participants used the Ask-the-Expert channel to pose individual questions to the smoking cessation expert on the consumption of nicotine-containing products.

#### Program Evaluation

**Figure 4.**
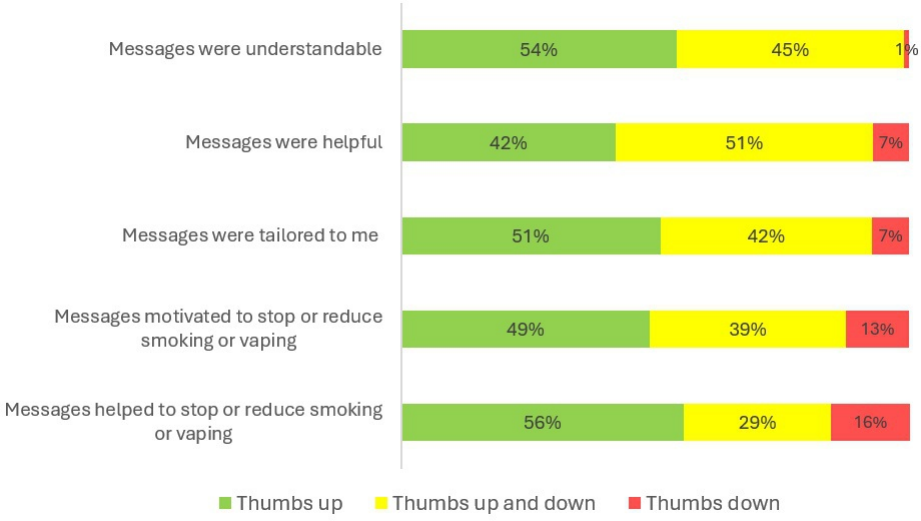
Evaluation of the coaching messages of the SmokeFreeCoach program (n=96).

Data on the evaluation of the program messages were available for 96/108 follow-up survey respondents. This is due to the fact that participants who reported that they usually had not read the program messages were not asked to rate them. Of the 96 participants, 52 (54%) rated the comprehensibility of the content positively, 40 (42%) stated that the program messages were helpful, 49 (51%) stated that the tips and information contained in the program messages were tailored to their personal situation, 47 (49%) stated that the program messages motivated them to smoke or vape less or to quit, and 54 (56%) stated that the program messages helped them to smoke or vape less or to quit. The results on the evaluation of the program messages are also displayed in Figure 4.

**Figure 5.**
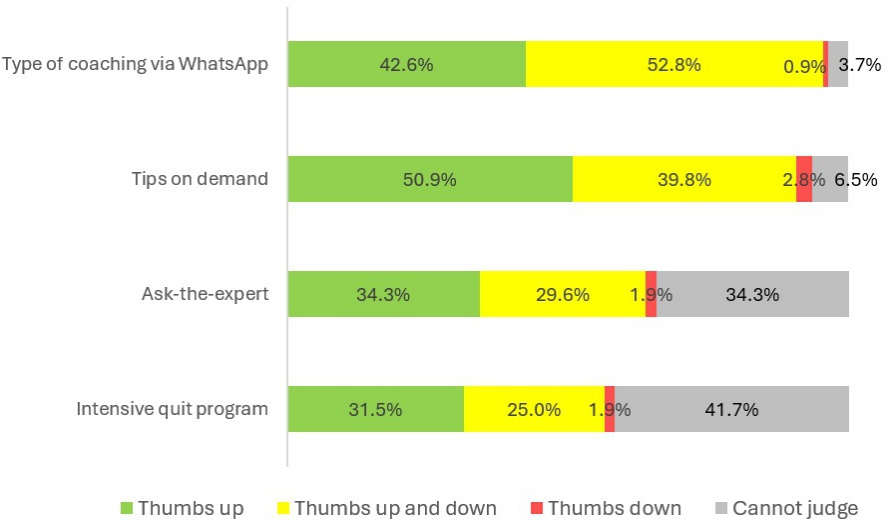
Evaluation of specific program elements of the SmokeFreeCoach (n=108).

Concerning the evaluation of specific program elements, we had data from all participants who completed the follow-up survey (n=108). The form of coaching via WhatsApp was rated positively by 46 (42.6%) participants, and 55 (50.9%) participants liked the available tips on demand. Overall, 36 (34.3%) participants rated the option to ask questions to an expert positively, and 34 (31.5%) participants rated the intensive quit program positively. The percentages of respondents who rated these program elements negatively were very low, ranging from 0.9% to 2.8%. The results concerning the evaluation of specific program elements are also shown in Figure 5.

The 108 postsurvey participants gave the program an overall rating on a scale of 1-5 stars. The mean program rating was 3.5 (SD 0.8) stars.

We received valid open-ended responses from 65 participants to the question “What content or functions were particularly interesting or helpful for you?” in the follow-up survey. A total of 5 participants stated that all of the program’s content and functions were interesting and helpful to them. The responses of 17 participants indicated that the tips sent by the program and the tips that participants could access themselves, such as tips on dealing with stress and distraction tips, were particularly interesting and useful. The fact that the SmokeFreeCoach is a WhatsApp-based program and easily accessible was appreciated by 9 participants, while 4 participants praised the ability to ask questions to experts. A total of 5 participants found the planning of individual strategies helpful. Altogether, 8 participants found the content of the program as well as the way the content was delivered (eg, videos, links, and statistics) helpful and interesting. Finally, 21 participants expressed their appreciation for the regular messages and reminders, which enabled them to constantly engage with the topic.

For the question “How could we improve the program?” in the follow-up survey, we received open-ended responses from 45 participants, of which 3 indicated having technical difficulties during the course of the intervention. Of the people making specific suggestions, 11 wanted the program to be better tailored to their individual life situation. Deepening or expanding the topics covered (eg, by suggesting more strategies for distraction during moments of craving, or by providing more information about the consequences of consuming nicotine or tobacco and also about other products, such as smokeless tobacco products) was suggested by 19 respondents. Four articipants expressed the wish that they could choose for themselves how many messages one gets and that the statements should be shortened in length. The need to avoid repetition of messages and content was also mentioned by 4 respondents. In addition, 3 participants specifically asked for a monitoring system with which they could track their daily cigarette use and earn credits. Finally, 1 participant reported having to click on links within the program to receive additional information as a barrier. Another one would have liked the expert to be available more often.

### Preliminary Program Efficacy

#### Days With Tobacco Cigarette Consumption

The GEE analyses revealed a statistically significant decrease in the number of days in the last 30 days on which tobacco cigarettes were smoked between baseline and the follow-up survey (incidence rate ratio [IRR] 0.68, *P*<.001). Considering only the data from participants who completed the follow-up survey, the average number of days in the last 30 days on which tobacco cigarettes were smoked decreased by 6.6 days from 20.6 (mean, SD 11.8) at baseline to 14.0 (mean, SD 12.0) at follow-up (Table 3).

**Table 3.** Pre-post comparison of indicators of nicotine use. The first 3 columns show the pre-post comparisons of the mean values, whereby only the data of the participants in the follow-up survey (n=108) were taken into account. The last 2 columns summarize the main results of the generalized estimating equation (GEE) analyses.

Indicator of nicotine use	Baseline mean (SD)	Follow-up mean (SD)	Mean value difference	IRR^a^ (95% CI)	*P* value
Number of days of tobacco cigarette consumption in the last 30 days	20.6 (11.8)	14.0 (12.0)	–6.6	0.68 (0.59-0.77)	<.001
Average number of tobacco cigarettes consumed per day in the last 30 days	7.2 (6.8)	4.1 (6.4)	–3.1	0.57 (0.45-0.73)	<.001
Number of days using electronic nicotine products in the last 30 days	11.1 (13.1)	7.7 (11.3)	–3.4	0.71 (0.55-0.90)	.005

a IRR: incidence rate ratio.

#### Quantity of Tobacco Cigarettes Smoked Per Day

Concerning the average number of tobacco cigarettes consumed per day in the last 30 days, the GEE analyses revealed a statistically significant decrease between the baseline and the follow-up survey (IRR 0.57, 95% CI 0.45-0.73; *P*<.001). Considering only the data from participants who completed the follow-up survey, the average number of tobacco cigarettes consumed per day in the last 30 days decreased by 3.1 cigarettes per day from 7.2 (mean, SD 6.8) at baseline to 4.1 (mean, SD 6.4) at follow-up (Table 3).

#### Days With Electronic Cigarette Consumption

Concerning the number of days using electronic nicotine products in the last 30 days, the GEE analyses also revealed a statistically significant decrease between the baseline and the follow-up survey (IRR 0.71, 95% CI 0.55-0.90; *P*<.005). Considering only the data from participants who completed the follow-up survey, the average number of days on which electronic nicotine products were used decreased by 3.4 days from 11.1 (mean, SD 13.1) days at baseline to 7.7 (mean, SD 11.3) days at follow-up (Table 3).

#### 30-Day Point Prevalence Abstinence

A total of 6/108 (5.6%) participants in the follow-up survey stated that they had not consumed either conventional cigarettes or electronic nicotine products in the last 30 days and could be considered as abstinent for the last 30 days at follow-up.

## Discussion

### Principal Findings

The study revealed three main findings: (1) the automated provision of individually tailored messages to reduce or stop smoking or vaping via WhatsApp was feasible, (2) the evaluation and use of the program in the target group of young people between the ages of 16 and 30 years was acceptable, and (3) the program may support cessation and reduction of tobacco cigarettes and electronic nicotine products.

The overall use and evaluation of the intervention program was acceptable. None of the 167 participants actively unsubscribed from the program or withdrew from study participation. On average, participants actively interacted in 5.5 (SD 3.5) of 11 program weeks with an average of 26.8 (SD 26.1) interactions over a period of 45.0 (SD 31.1) days. A comparison of these results with findings on program engagement in other digital programs developed specifically for young people shows that participation in the SmokefreeCoach program is comparable or even higher. For example, young people participating in a text messaging–based smoking cessation program in Switzerland responded to an average of 6.5 (SD 3.7) of 11 SMS text messaging questions asked [30]. Within a multiple behavior prevention program provided via smartphone app and aimed at Swiss adolescents, the average number of dialogs completed with a conversational agent was 2.1 (SD 3.5) out of 16 [31]. In an app-based alcohol prevention program for young people in Switzerland, an average of 3.8 (SD 3.6) out of 10 dialogs with a conversational agent were completed, and the average duration of program use was 38.3 (SD 52.1) days [32]. Adolescents who registered for the Australian health promotion app Health4life were active for an average of 9.7 (SD 18.8) days [33].

The vast majority of respondents expressed positive or at least neutral evaluations of the program, the program messages, and the program elements. However, the answers to the question “How can we improve the program?” indicate that there is room for improvement. Among the respondents’ suggestions for improving the program, better tailoring of the program content to the respective person, and expanding the program to include information on untreated topics, such as substitution of tobacco cigarettes with other nicotine-containing products and on nicotine products that are not included, such as smokeless tobacco products, stood out in particular.

Although the results on the preliminary efficacy of the intervention program are derived from a pre-post study, they suggest that the SmokeFreeCoach could be a promising tool for supporting smoking and vaping cessation among adolescents and young adults. Throughout the study, a significant reduction was observed in 3 indicators of cigarette and nicotine consumption, and 6 participants stopped smoking.

As there are hardly any low-threshold evidence-based programs to support young people in quitting smoking or vaping [15], it seems sensible to pursue this intervention approach further on the basis of the results obtained. Although text messaging–based interventions are among the most promising digital smoking and vaping cessation interventions for young people [15], it is questionable whether such good results can be achieved in the future as, for example, in the study from Graham et al [34] or Ybarra et al [35], in view of the increasingly reduced use and limited possibilities of this channel. Communication via instant messenger apps like WhatsApp or Viber (Rakuten) has largely replaced SMS text messaging and offers more options for integrating media content and also for combining personal and automated modes of communication.

Compared to cessation apps, for example, [36], which have to be installed and opened separately, messenger apps offer the advantage that they are already available and are used regularly. Therefore, their access is likely to be lower-threshold and program use higher, which is indicated by the results of this study. On the other hand, the now numerous features of WhatsApp, such as “channels,” “communities,” or the recently integrated “Meta AI,” also pose the risk that messages are more likely to be ignored within WhatsApp intervention programs than in app-based applications developed solely for this purpose.

### Limitations

This study has several limitations:

1. The longitudinal one-group pre-post design does not allow sound conclusions on the effectiveness of the program.
2. The reported outcomes on tobacco smoking and nicotine use are based on self-reports and were not verified using biochemical parameters. However, numerous studies have shown that self-reports on nicotine consumption are mostly valid; especially when delivered online and anonymously, they are not subject to strong bias [37].
3. The results of the attrition analysis reveal significant differences in the use of the program between those who participated in the follow-up survey and those who did not. Therefore, a bias toward those who found the program effective and acceptable is likely.

### Outlook

In view of the promising results of this study, it seems crucial to conduct a randomized controlled trial in order to draw sound conclusions on the effectiveness of the program. Based on the suggestions for improvement made by the participants in this study and the increasing popularity of smokeless nicotine products, such as snus or nicotine pouches among young people [38], it seems reasonable to expand the program to include coaching content also addressing this variant of nicotine use.

### Conclusions

This is the first study that investigated the acceptability and preliminary efficacy of an automated WhatsApp-based program to support smoking and vaping cessation. Its results suggest that the automated provision of individually tailored coaching messages via WhatsApp is feasible and the SmokefreeCoach program was modestly accepted by the target group of young people. The preliminary results on program efficacy suggest that the program could provide a low-cost and scalable measure to decrease the prevalence rate of nicotine use. Overall, the results of this study, the promising efficacy of SMS text messaging and WeChat-based smoking cessation programs [17,23,37], and the popularity of instant messaging apps [24] suggest that WhatsApp-based interventions are a promising means of helping young people to quit smoking and vaping.

## Supplementary material

10.2196/65301 Multimedia Appendix 1 Baseline- and follow-up assessments.

## Abbreviations

API: application programming interface
e-cigarette: electronic cigarette
GDPR: General Data Protection Regulation
GEE: generalized estimating equation
IRR: incidence rate ratio
ISGF: Institut für Sucht- und Gesundheitsforschung
ZFPS: Zürcher Fachstelle zur Prävention des Suchtmittelmissbrauchs

## References

[1] GBD 2019 Tobacco Collaborators (2021). Study, spatial, temporal, and demographic patterns in prevalence of smoking tobacco use and attributable disease burden in 204 countries and territories, 1990-2019: a systematic analysis from the Global Burden of Disease Study 2019. Lancet.

[2] Jakob J, Cornuz J, Diethelm P (2017). Prevalence of tobacco smoking in Switzerland: do reported numbers underestimate reality?. Swiss Med Wkly.

[3] West R (2017). Tobacco smoking: health impact, prevalence, correlates and interventions. Psychol Health.

[4] (2025). Tobacco consumption (age: 15+). Swiss Monitoring System of Addiction and Noncommunicable Diseases.

[5] Pirie K, Peto R, Reeves GK, Green J, Beral V, Million Women Study Collaborators (2013). The 21st century hazards of smoking and benefits of stopping: a prospective study of one million women in the UK. Lancet.

[6] Consumption of e-cigarettes (age: 15+) [Web page in German]. Swiss Monitoring System of Addiction and Noncommunicable Diseases.

[7] Lindson N, Butler AR, McRobbie H (2024). Electronic cigarettes for smoking cessation. Cochrane Database Syst Rev.

[8] Hartmann-Boyce J, Butler AR, Theodoulou A (2023). Biomarkers of potential harm in people switching from smoking tobacco to exclusive e-cigarette use, dual use or abstinence: secondary analysis of Cochrane systematic review of trials of e-cigarettes for smoking cessation. Addiction.

[9] Hammond D, Reid JL, Goniewicz ML (2025). Nicotine exposure from smoking tobacco and vaping among adolescents. JAMA Netw Open.

[10] Anderer S (2025). Rise in vaping keeps tobacco use high. JAMA.

[11] Banks E, Yazidjoglou A, Brown S (2023). Electronic cigarettes and health outcomes: umbrella and systematic review of the global evidence. Med J Aust.

[12] Hartmann-Boyce J, Chepkin SC, Ye W, Bullen C, Lancaster T (2018). Nicotine replacement therapy versus control for smoking cessation. Cochrane Database Syst Rev.

[13] Hartmann-Boyce J, Livingstone-Banks J, Ordóñez-Mena JM (2021). Behavioural interventions for smoking cessation: an overview and network meta-analysis. Cochrane Database Syst Rev.

[14] Wu AD, Lindson N, Hartmann-Boyce J (2022). Smoking cessation for secondary prevention of cardiovascular disease. Cochrane Database Syst Rev.

[15] Park E, Zhou Y, Chen C, Chacko T, Mahoney M, Chang YP (2023). Systematic review: interventions to quit tobacco products for young adults. BMC Public Health.

[16] Thrul J, Stemmler M, Goecke M, Bühler A (2015). Are you in or out? Recruitment of adolescent smokers into a behavioral smoking cessation intervention. Addict Behav.

[17] Zhou X, Wei X, Cheng A (2023). Mobile phone-based interventions for smoking cessation among young people: systematic review and meta-analysis. JMIR Mhealth Uhealth.

[18] Jakob R, Harperink S, Rudolf AM (2022). Factors influencing adherence to mHealth apps for prevention or management of noncommunicable diseases: systematic review. J Med Internet Res.

[19] Guo YQ, Chen Y, Dabbs AD, Wu Y (2023). The effectiveness of smartphone app-based interventions for assisting smoking cessation: systematic review and meta-analysis. J Med Internet Res.

[20] Luo T, Li MS, Williams D (2021). Using social media for smoking cessation interventions: a systematic review. Perspect Public Health.

[21] Sweileh WM (2024). Technology-based interventions for tobacco smoking prevention and treatment: a 20-year bibliometric analysis (2003-2022). Subst Abuse Treat Prev Policy.

[22] Ramo DE, Thrul J, Delucchi KL (2018). A randomized controlled evaluation of the tobacco status project, a Facebook intervention for young adults. Addiction.

[23] Tang J, Yang J, Liu Y (2023). Efficacy of WeChat-based online smoking cessation intervention ('WeChat WeQuit’) in China: a randomised controlled trial. EClinicalMedicine.

[24] Külling C (2022). JAMES: jugend, aktivitäten, medien - erhebung schweiz [Report in German]. https://www.zhaw.ch/storage/psychologie/upload/forschung/medienpsychologie/james/2018/Bericht_JAMES_2022_de.pdf.

[25] Prochaska JO, Velicer WF (1997). The transtheoretical model of health behavior change. Am J Health Promot.

[26] McAlister AL, Perry CL, Parcel GS, Practice KG, Rimer BK, Viswanath K (2008). Health Behavior and Health Education: Theory, Research.

[27] Mair JL, Salamanca-Sanabria A, Augsburger M (2023). Effective behavior change techniques in digital health interventions for the prevention or management of noncommunicable diseases: an umbrella review. Ann Behav Med.

[28] Zeger SL, Liang KY, Albert PS (1988). Models for longitudinal data: a generalized estimating equation approach. Biometrics.

[29] Mayring P (2010). Qualitative Content Analysis: Fundamentals and Techniques.

[30] Haug S, Schaub MP, Venzin V, Meyer C, John U (2013). Efficacy of a text message-based smoking cessation intervention for young people: a cluster randomized controlled trial. J Med Internet Res.

[31] Haug S, Boumparis N, Wenger A, Schaub MP, Paz Castro R (2022). Efficacy of a mobile app-based coaching program for addiction prevention among apprentices: a cluster-randomized controlled. Int J Environ Res Public Health.

[32] Haug S, Boumparis N, Wenger A, Paz Castro R, Schaub MP (2023). Mobile app-based coaching for alcohol prevention among adolescents: pre-post study on the acceptance and effectiveness of the program “MobileCoach Alcohol”. Int J Environ Res Public Health.

[33] Thornton L, Brown HM, Osman B (2022). Factors associated with adolescents’ engagement with a Healthy Lifestyles app. Procedia Comput Sci.

[34] Graham AL, Amato MS, Cha S, Jacobs MA, Bottcher MM, Papandonatos GD (2021). Effectiveness of a vaping cessation text message program among young adult e-Cigarette users: a randomized clinical trial. JAMA Intern Med.

[35] Ybarra ML, Holtrop JS, Prescott TL, Rahbar MH, Strong D (2013). Pilot RCT results of Stop My Smoking USA: a text messaging–based smoking cessation program for young adults. Nicotine Tob Res.

[36] Baskerville NB, Struik LL, Guindon GE (2018). Effect of a mobile phone intervention on quitting smoking in a young adult population of smokers: randomized controlled trial. JMIR Mhealth Uhealth.

[37] Fang YE, Zhang Z, Wang R (2023). Effectiveness of eHealth smoking cessation interventions: systematic review and meta-analysis. J Med Internet Res.

[38] Patel M, Kierstead EC, Kreslake J, Schillo BA (2023). Patterns of oral nicotine pouch use among U.S. adolescents and young adults. Prev Med Rep.

