# Supplementary material for: WhatsApp-Based Coaching Program to Support Smoking and Vaping Cessation Among Young People: Pre-Post Study on Acceptance and Preliminary Efficacy
Source: JMIR Mhealth Uhealth. 2025 Oct 2;13:e65301. doi: 10.2196/65301 (PMC12490768; doi:10.2196/65301)
Supplement: Multimedia Appendix 1 [file mhealth-v13-e65301-s001.docx]

**Screening items**

**How old are you?**

_____ years

**How often have you used nicotine-containing products such as tobacco cigarettes, e-cigarettes (vapes) or shisha in the last 30 days?**

□ Every day

□ Not every day, but at least once a week

□ Less than once a week

□ Never

**Baseline Survey**

**Please choose a user name with which we can address you within the program** (you can use a nickname or your nickname, for example).

_______________

**Are you...**

□ Male

□ Female

□ Diverse

**In which canton do you currently have your main residence?**

□ Zurich

□ Aargau

□ St.Gallen

□ Grisons

□ Other canton

□ Other country

**How did you first hear about the *SmokefreeCoach*?**

□ Social Media

□ Advertising in daily/commuter newspaper

□ Friends/acquaintances

□ School/University

□ At work

**How many days have you smoked tobacco cigarettes in the last 30 days?**

On ____days

**How many tobacco cigarettes do you usually smoke in a day when you smoke?**

____ cigarettes/day

**On how many days have you used electronic nicotine products (e.g. vapes, e-shisha, puff bars, IQOS) in the last 30 days?**

On ____days

**Which statement best applies to you?**

Over the next 10 weeks, I would like to....

□ ...stop smoking/vaping

□ ...smoke/vape less

□ ...neither quit nor smoke/vape less

**In order to stop or reduce smoking and vaping, it is helpful to know the emotional states in which you smoke/vape frequently.**

Choose 1 to 3 emotional states that most often lead you to smoke/vape.

When I...

□ ...am bored

□ ...am excited or nervous

□ ...am sad

□ ...am angry

□ ...am in a good mood

□ ...would like to have a moment to myself

□ ...looking for contact with others

□ ...would like to reward myself

□ ...would like to relax

□ ...want to feel safer

**Now choose 1-3 situations in which you smoke or vape particularly often**

□ When I go out

□ When I drink alcohol

□ After the meal

□ When drink coffee / energy drinks

□ In the car

□ When I'm waiting somewhere

□When I have a break

□ After having sex

□ Before I go to bed

□ When I leave the house in the morning

□When I talk to others or on the phone

□ When someone offers me a cigarette

Is there another situation in which you smoke/vape particularly often? If so, you can enter it in the following field:

______ I smoke/vape particularly often.

(e.g. when I am *studying* for an *exam ...*)

***For participants with a reduction goal:***

In order to smoke or vape less, it is helpful to first stop using nicotine products in certain situations. Now choose situations in which you no longer want to smoke or vape. If you choose situations in which you smoke or vape frequently, you can significantly reduce your consumption of nicotine-containing products. However, you can also start by choosing situations in which it is particularly easy for you to stop.

**Now choose 1-3 situations in which you no longer want to smoke/vape in the future.**

□ When I drink coffee / energy drinks

□ When I'm out

□ When I drink alcohol

□ After the meal

□ In the car

□ When I'm waiting somewhere

□ When I take a break

□ After sex

□ Before I go to bed

□ When I leave the house in the morning

□ When I talk to others or on the phone

□When someone offers me a cigarette

**What is bothering you?**

*Smoking and vaping is sometimes associated with stressful situations. Now choose up to 3 things that are currently bothering you.*

□ Pressure to perform

□ Lack of time

□ Poor self-confidence/self-doubt

□ Psychological problems

□ Arguments with family/ parents/ friends

□ Feeling of being an outsider

□ Loneliness

□ Missing or existing romantic relationship

□ Thinking about what I will do in the future (e.g. career and study choices)

□ Concern due to current crises (e.g. corona, war, global warming)

**How important are the following benefits of not smoking/vaping to you?**

Choose 1 to 3 reasons that are most important to you.

If I stop or reduce smoking/vaping...

□ ...I have better fitness and stamina
□ ...I am doing something for my health
□ ...I feel freer and more independent
□ ...I have more time
□ ...I have more money for other things
□ ...I smell better (clothes, breath)
□ ...I have higher fertility
□ ...I contribute to less child labor and environmental pollution

Perhaps you also have a very important personal reason:

If I stop or reduce smoking/vaping, _________________

**What concerns do you have about quitting or reducing smoking/vaping?**

If I stop or reduce smoking/vaping...

□ ...I could gain weight

□ ...I could feel excluded among my friends

□ ...I could get restless and nervous

□ ...I could no longer relax so well

□ ...I would have less fun going out

□ ...I would be less awake/concentrated

□ ...I could reward myself less well

□ ...I could enjoy certain situations less

Thank you for your details! This will help us to tailor the coaching to you over the next few weeks.

**Follow up survey**

Let`s start with a few questions about your nicotine use...

**How many days have you smoked tobacco cigarettes in the last 30 days?**

On ____ days

**How many tobacco cigarettes do you usually smoke per day when you smoke?**

____ cigarettes/day

**On how many days have you used electronic nicotine products (e.g. vapes, e-shisha, puff bars, IQOS) in the last 30 days?**

On ____ days

Let`s continue with some questions about the *SmokefreeCoach* program

**The smokefreeCoach regularly gave you tips and information on smoking/vaping, quitting or reducing nicotine use and dealing with stress. Have you watched or read these messages?**

□ Yes, I usually read them thoroughly

□ Yes, I usually looked at them briefly

□ No, I have not read them

**How do you rate the coaching messages from the SmokefreeCoach?**

1. The content was easy to understand
2. The tips and information were helpful for me
3. The tips and information were tailored to my personal situation
4. The messages motivated me to smoke/vape less or to quit
5. The messages helped me to smoke/vape less or to quit

*Answer options in each case: thumbs up, thumbs up/down, thumbs down*

**How would you rate the following elements of the SmokefreeCoach?**

|  | Thumbs up | Thumbs up/down | Thumbs down | I cannot judge |
| --- | --- | --- | --- | --- |
| The form of coaching via WhatsApp | □ | □ | □ | □ |
| The available tips on motivation/dealing with stress and cravings | □ | □ | □ | □ |
| The opportunity to ask an expert questions | □ | □ | □ | □ |
| The higher intensive quit program | □ | □ | □ | □ |

**Which content or functions were particularly interesting or helpful for you?**

________________________

**How could we improve the program?**

________________________

**How many stars would you give the program (1-5)?**

________________________

Thank you very much for your opinion and your information! These are very important for the optimization of the SmokefreeCoach.
